# Specific micro-RNA expression patterns distinguish the basal and luminal subtypes of muscle-invasive bladder cancer

**DOI:** 10.18632/oncotarget.13284

**Published:** 2016-11-10

**Authors:** Andrea E. Ochoa, Woonyoung Choi, Xiaoping Su, Arlene Siefker-Radtke, Bogdan Czerniak, Colin Dinney, David J. McConkey

**Affiliations:** ^1^ Department of Urology, The University of Texas MD Anderson Cancer Center, Houston, Texas, USA; ^2^ Department of Cancer Biology, The University of Texas MD Anderson Cancer Center, Houston, Texas, USA; ^3^ Department of Bioinformatics, The University of Texas MD Anderson Cancer Center, Houston, Texas, USA; ^4^ Department of Pathology, The University of Texas MD Anderson Cancer Center, Houston, Texas, USA; ^5^ Department of Genitourinary Medical Oncology, The University of Texas MD Anderson Cancer Center, Houston, TX, USA; ^6^ Program in Experimental Therapeutics, University of Texas Graduate School of Biomedical Sciences, Houston, Texas, USA; ^7^ Program in Cancer Biology, University of Texas Graduate School of Biomedical Sciences, Houston, Texas, USA; ^8^ Johns Hopkins Greenberg Bladder Cancer Institute, Baltimore, MD, USA

**Keywords:** urothelial cancer, TCGA, consensus clustering, PPARG, EMT

## Abstract

The roles of non-coding RNAs in controlling clinical and biological heterogeneity in bladder cancer remain unclear. We used TCGA's published dataset (n = 405 tumors) as a discovery cohort and created a new validation cohort to define the miRNA expression patterns in the basal and luminal molecular subtypes of muscle-invasive bladder cancer (MIBC). We identified 63 miRNAs by PAM, which optimally identified basal and luminal tumors. The targets of the top luminal miRNAs were activators of EMT (ZEB1, ZEB2) and basal subtype transcription (IL-6, EGFR, STAT3), whereas the targets of the top basal miRNAs were involved in adipogenesis pathways and luminal breast cancer (ERBB2, ERBB3). We also identified a 15-miRNA signature that identified stromally infiltrated basal and luminal MIBCs corresponding to the “cluster IV/immune undifferentiated/claudin-low” and “cluster II/luminal immune” subtypes identified previously, which likely contain samples with higher infiltration rates. Using the 63-miRNA signature, we accurately assigned MIBCs to the basal and luminal subtypes and confirmed that patients with basal tumors had shorter overall survival. The results strongly suggest that miRNAs contribute to the control of the gene expression patterns observed in basal and luminal MIBCs and that they can be used as biomarkers and candidate therapeutic targets.

## INTRODUCTION

Muscle-invasive bladder cancer (MIBC) is the fourth most common cancer type in men in the United States, occurring less frequently in women. It is a highly heterogeneous disease in which approximately half of patients are cured by surgery with or without cisplatin-based chemotherapy, while the other half succumb to rapid disease progression [1]. Frontline treatment regimens had not changed for decades until anti-PDL1 immune checkpoint blockade was approved very recently [2, 3], and prognostication is still based on clinical and pathological criteria. Fortunately, several recent large-scale genomics projects have provided new insights into the molecular heterogeneity of MIBCs that likely influences clinical heterogeneity. Whole genome mRNA expression profiling by several independent research groups demonstrated that MIBCs can be subdivided into molecular subtypes that share biomarkers with the intrinsic basal and luminal subtypes of breast cancers [4-7]. Cancers in one subtype (termed “SCC-like” or “basal”) [8] were associated with advanced and metastatic disease at presentation and shorter disease-specific and overall survival, whereas patients whose cancers belonged to another subtype (termed “papillary” or “luminal”) had better outcomes [4, 5, 7]. Basal cancers were enriched with squamous histopathological features [4, 5, 7], whereas luminal cancers were enriched with papillary histopathological features and activating DNA mutations and fusions involving fibroblast growth factor receptor-3 (FGFR3) [4, 5, 9]. Therefore, it appears that basal and luminal MIBCs have very distinct clinical and biological properties and therefore should be managed as distinct disease entities. As a consequence, there is strong interest in developing reliable clinical tools that can be used to accurately assign MIBCs to the basal and luminal subtypes.

Micro-RNAs (miRNAs) are a class of non-coding RNAs containing 19 to 24 nucleotides that control expression of their target mRNAs by inhibiting protein translation and promoting mRNA degradation. Micro-RNAs are attractive as cancer biomarkers because they are more stable than mRNAs in formalin-fixed, paraffin-embedded (FFPE) tissues and accessible body fluids [10, 11]. While miRNA expression has been explored in bladder cancer, most past studies focused on miRNAs that are differentially expressed in cancers as compared to normal urothelium, including miR-142, the miR-200 family, miR-100, and miR-99a [12]. We wondered whether miRNAs could also be used as biomarkers to identify the basal and luminal molecular subtypes of bladder cancer and possibly as novel therapeutic agents [13]. To test this hypothesis, we used TCGA's matched whole genome mRNA and miRNA expression data and generated new miRNA sequencing data on an independent cohort of 62 muscle-invasive tumors from our own institution to explore the patterns of miRNA expression in basal and luminal cancers.

## RESULTS

**Figure 1:**
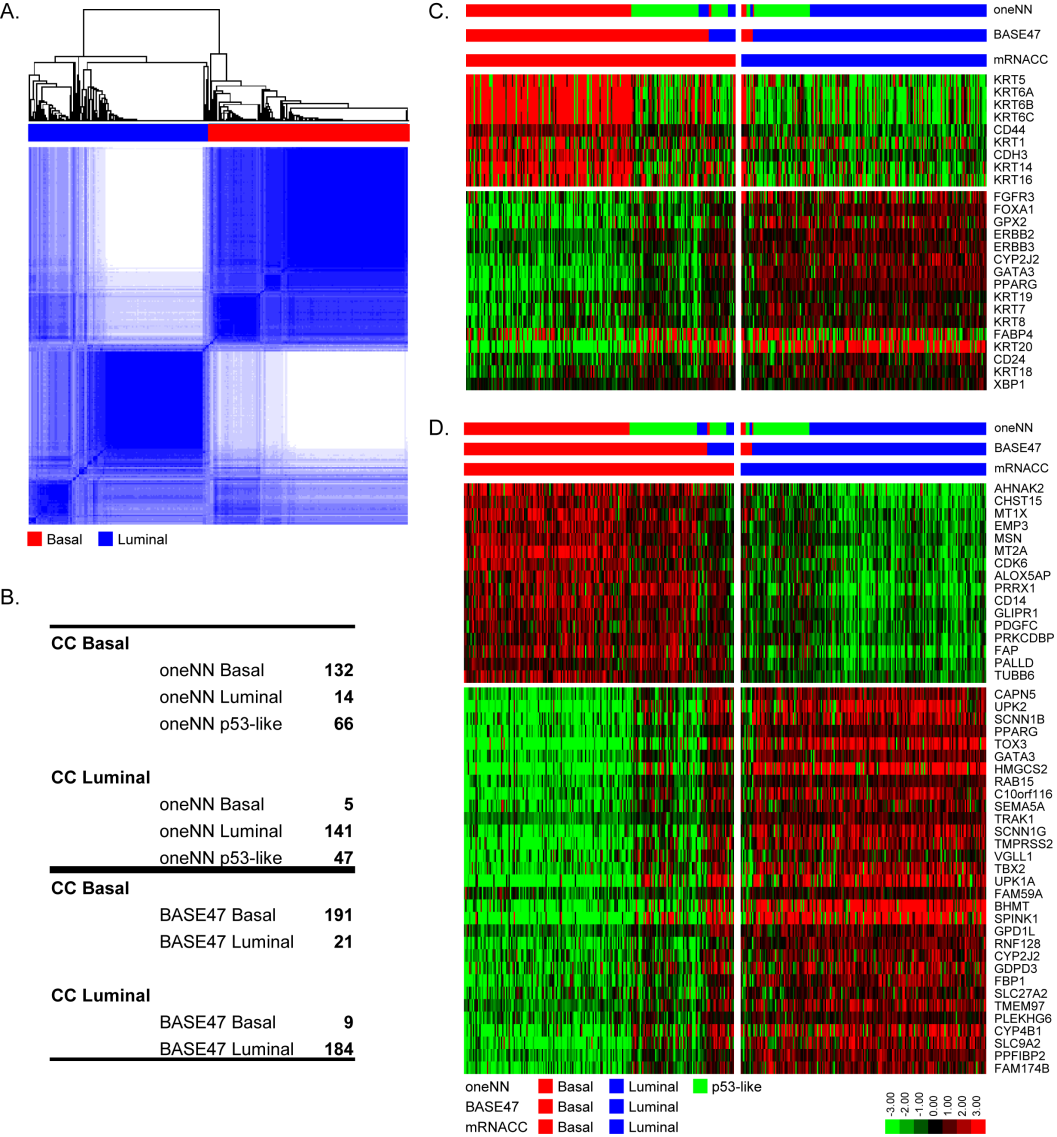
Validation of the basal and luminal subtypes mRNA CC was performed with TCGA's cohort (*n* = 405). **A.** mRNA CC (k = 2) solution. **B.** Comparison of mRNA CC subtype assignments to MDA oneNN assignments (top), and to BASE47 assignments (bottom). **C.** The heatmap depicts relative expression of MDA basal (top) and luminal (bottom) markers. **D.** The heatmap depicts relative expression of BASE47 basal (top) and luminal (bottom) markers.

The TCGA cohort is the largest high quality whole transcriptome MIBC dataset available at present (*n* = 405 MIBCs). We used the TCGA RNAseq data and unsupervised analyses, consensus clustering (CC), to determine whether previous conclusions about the molecular subtypes of bladder cancer were reproducible. Consistent with our previous conclusions [14], the results revealed that a three cluster (k = 3) solution best fit the data mathematically (Figure S1A), and the biomarkers that were enriched in each of the 3 CC subtypes overlapped significantly with those associated with the basal, p53-like, and luminal subtypes we had identified previously (Figure S1). However, the CC subtype assignments were only 75% identical to those made using our one-nearest neighbor (oneNN) classifier (Figure S1B) [14]. Most of the discrepancies were due to class switches between the p53-like and luminal tumors, consistent with past conclusions [14]. Furthermore, in spite of the fact that a three-cluster solution was optimal mathematically, we noted that basal and luminal biomarker expression was almost entirely mutually exclusive in the p53-like tumors (Fig S1C, S1D), suggesting that a two-cluster (k = 2) solution would be more biologically accurate. Consistent with this idea, using a k = 2 solution (Figure 1A), we observed excellent overlap (93%) with subtype assignments made using an independent basal/luminal (k = 2) PAM classifier (BASE47) [6], and when the oneNN p53-like tumors were omitted, we also observed an overlap of 93% with the oneNN basal and luminal subtype assignments (Figure 1B). Direct visualization of basal and luminal biomarkers confirmed good separation of the basal and luminal CC subtypes, although a fraction of the basal tumors, corresponding to some of the oneNN p53-like tumors, had noticeably lower expression of both basal and luminal biomarkers (Figure 1C/1D).

**Figure 2:**
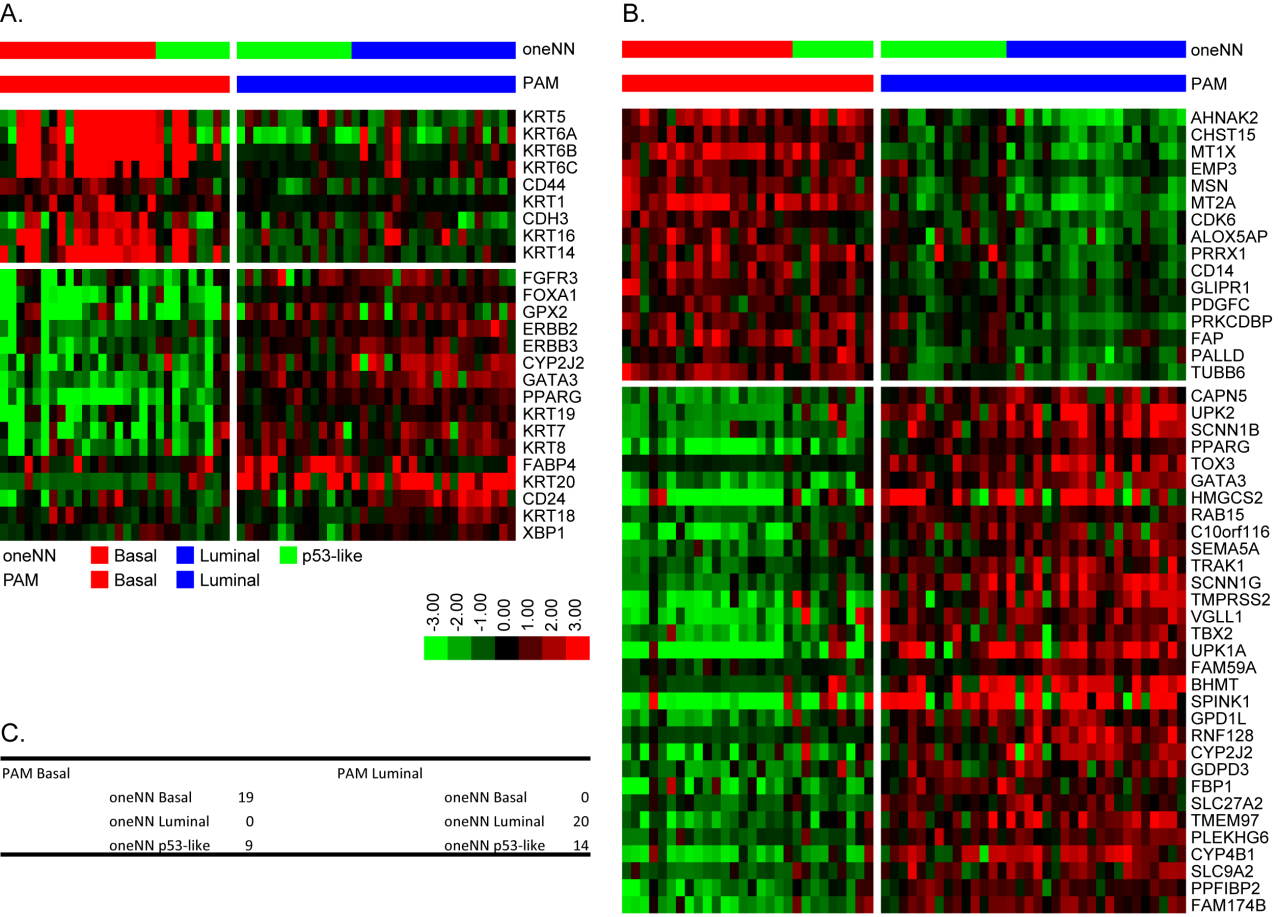
PAM identifies basal and luminal tumors in the 62 FF validation cohort **A.** The heatmap depicts relative expression of MDA basal (top) and luminal (bottom) markers as a function of PAM subtype assignment. **B.** The heatmap depicts relative expression of BASE47 basal (top) and luminal (bottom) markers as a function of PAM subtype assignment. **C.** Comparison of mRNA PAM subtype assignments and MDA oneNN assignments.

We used the TCGA mRNA dataset and the k = 2 CC subtype assignments as a training set to develop our own mRNA PAM classifier, using 62 samples from the MDA fresh frozen (FF) cohort (GSE48075) for validation (Table S1). We used 12,407 mRNAs that passed filtering criteria in TCGA's cohort and were present in the FF cohort to develop the PAM classifier, which resulted in a solution that contained 593 mRNAs that contained 39 of the 47 BASE47 genes (Figure S2A). We noted a few discrepancies in the subtype assignments made using this new PAM classifier and BASE47, which were largely due to the presence of a small fraction of tumors that were double positive for basal and luminal biomarkers (Figure S2). The 593 PAM mRNAs were then used to make subtype predictions in the 62 FF cohort (Figure S2B), resulting in 28 basal tumors and 34 luminal tumors. The 28 predicted basal tumors encompassed all of the basal tumors originally identified by oneNN prediction and had high expression of basal markers and basal BASE47 genes. The 34 predicted luminal tumors encompassed all of the luminal tumors identified by oneNN prediction and had high-level expression of PAM luminal markers and luminal BASE47 genes (Figure 2).

**Figure 3:**
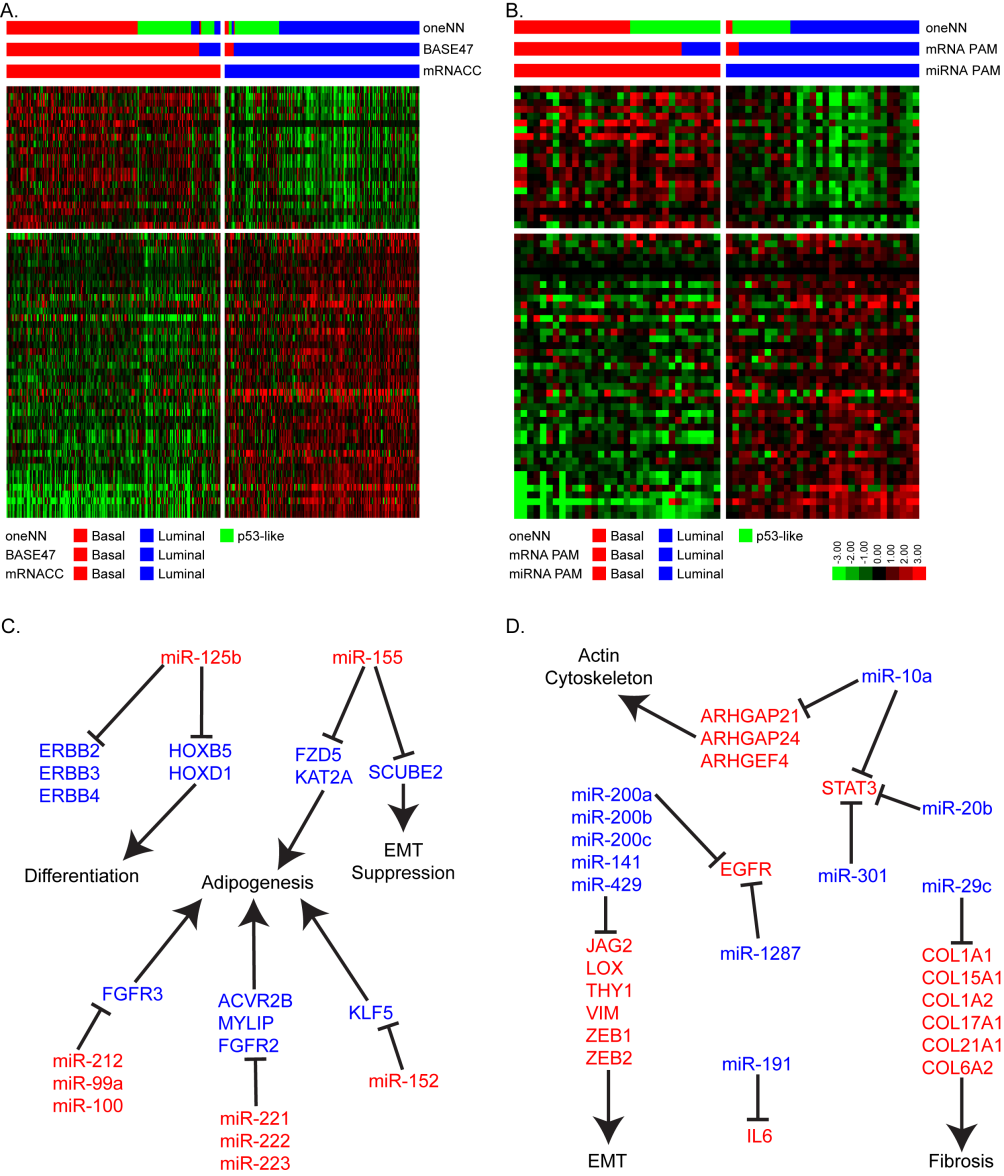
Micro-RNA PAM identifies basal and luminal tumors **A.** The heatmap depicts relative expression of 63 miRNAs identified by PAM to accurately assign basal and luminal tumors in TCGA's cohort (*n* = 405). **B.** The heatmap depicts relative expression of the 63-miRNA predictor in the FF cohort (*n* = 62). **C.** Schematic representation of basal miRNAs and their luminal associated mRNA target genes and pathways. **D.** Schematic representation of luminal miRNAs and their basal associated mRNA target genes and pathways.

After identifying the basal and luminal MIBCs in the two datasets, we characterized their differential miRNA expression patterns as a step toward identifying a miRNA-based signature that could distinguish them. We first used the same unbiased approach we applied to TCGA's mRNA dataset, this time using TCGA's miRNAseq data from the 405 MIBCs (Figure S3). The results were consistent with a k = 2 solution (Figure S3A) and revealed 77% and 73% concordance with the basal/luminal subtype assignments made by mRNA consensus clustering and BASE47, respectively (Figure S3B). However, when we examined basal and luminal mRNA biomarker expression within the miRNA clusters, we observed that a significant fraction of PAM-defined luminal tumors clustered with the miRNA-defined basal cluster (Figure S3C/S3D). Therefore, we concluded that a supervised approach based on the mRNA subtype calls would generate more accurate calls. Because no validation cohort consisting of matched mRNA and miRNA expression profiling data was available, we generated a new one by performing small RNA sequencing (Ion Torrent platform) on our 62 FF validation cohort [14]. Using TCGA's cohort as a training set, we used 412 miRNAs that passed filtering criteria in TCGA's cohort and were present in the FF cohort to develop a miRNA PAM classifier, which resulted in a 63 miRNA solution (Figure 3A/3B). A survey of the results suggested that the known biological targets of these miRNAs were relevant to basal and luminal biology. Specifically, basal tumors expressed high levels of miR-155, miR-142, miR-221, miR-222 and miR-223, which are miRNAs commonly associated with aggressiveness in other solid tumors and poor prognosis [15-19]. The luminal tumors expressed high levels of all of the members of the miR-200 family (miR-200a/b/c, miR-141, miR-429), consistent with previous findings [5, 14]. Members of the miR-200 family are inhibitors of epithelial-mesenchymal transition (EMT) that directly target ZEB1 and ZEB2, core EMT transcription factors that directly inhibit transcription of the “epithelial” adhesion molecule E-cadherin that are highly expressed in basal tumors [20]. Luminal tumors express high levels of E-cadherin and low levels of ZEB1/2, consistent with the idea that members of the miR-200 family play important roles in controlling their biological properties. We proceeded to confirm that these 63 miRNAs could accurately distinguish basal and luminal tumors using hierarchical clustering (Figure S4). This resulted in 85% and 83% concordance with the subtype assignments assigned by mRNA consensus clustering and BASE47, respectively.

We then used the Ingenuity Pathway Analysis (IPA) miRNA target filter and miRTarBase to identify additional miRNA-mRNA relationships that were either experimentally observed or were highly predicted in the TargetScan database. The results revealed that many of the basal subtype-associated miRNAs targeted mRNAs involved in adipogenesis, differentiation, and EMT suppression (Figure 3C). Specifically, several basal miRNAs (miR-125b, miR-142, miR-143, miR-152, miR-155, and miR-221/222/223) have been predicted to block genes in the adipogenesis and RXR activation pathways [21-23]. Most notably, miR-125b, miR-223, miR-99a, and miR-212 target FGFR2 and FGFR3, which are involved in luminal MIBC biology [5, 22, 24, 25]. Several basal miRNAs (miR-125b, miR-142, miR-152, miR-146b, miR-222, and miR-212) have also been predicted to target luminal factors that have been previously identified in breast cancer, including ERBB2, ERBB3, ERBB4, and FOXA1 [26-28]. MiR-125b has also been shown to inhibit homeobox (HOX) genes, which control urothelial terminal differentiation [29] and are highly expressed by luminal bladder cancers.

Likewise, the miRNAs that were enriched in luminal MIBCs target pathways associated with invasion and metastasis (EMT, fibrosis, and the actin cytoskeleton) (Figure 3D). For example, experimentally observed targets of miR-29c include 6 different collagens that are associated with fibrosis and possibly support cancer-associated fibroblast infiltration [30, 31]. MiR-10a, miR-20b and miR-301 are also predicted to target STAT3, while miR-1287 and miR-191 are predicted to target EGFR and IL6, respectively. EGFR and IL6 are upstream regulators of STAT3, and all have been implicated in basal breast and bladder cancers [4, 32].

**Figure 4:**
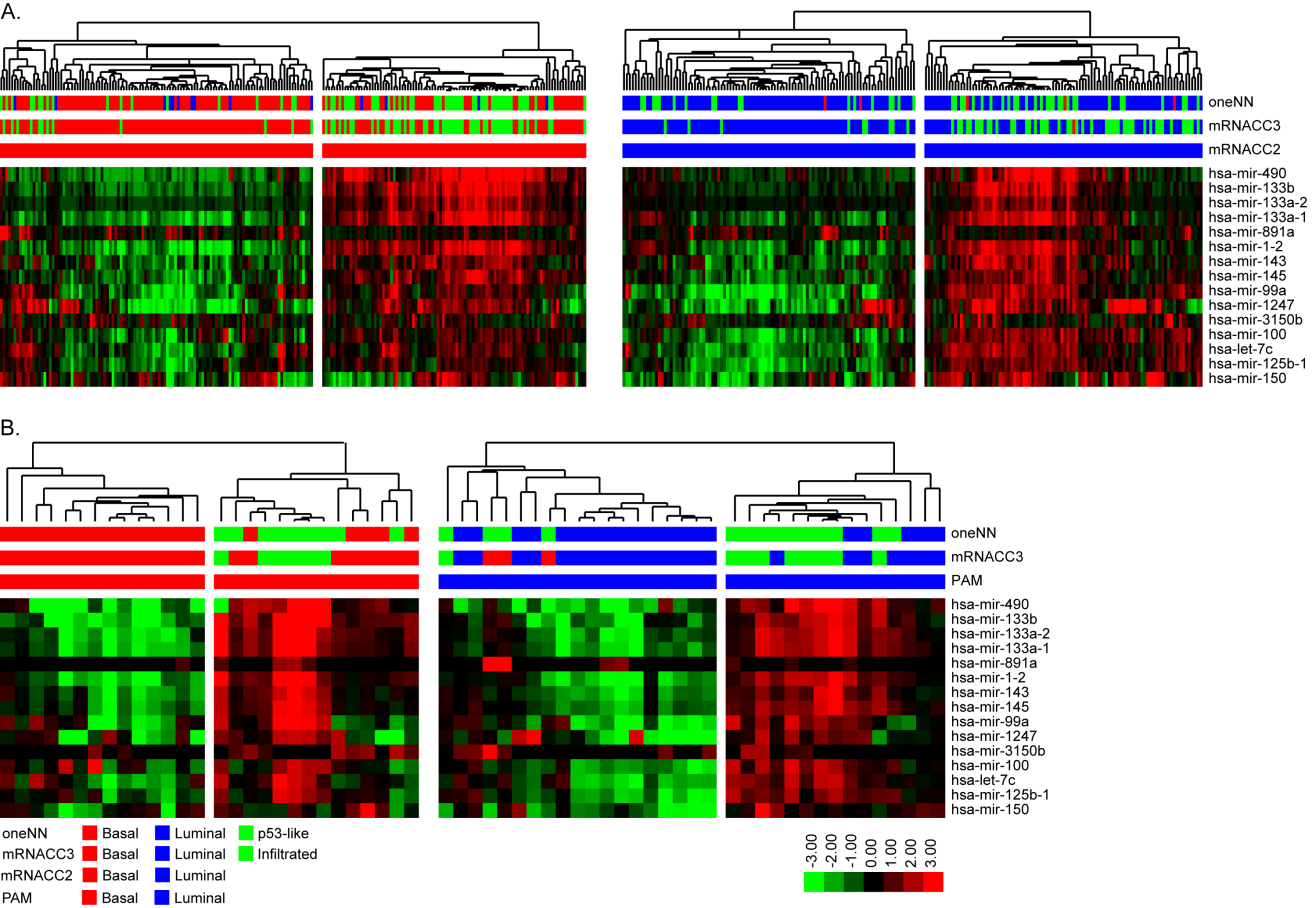
Differential expression analysis identified 15 miRNAs that define infiltrated/p53-like subsets of the basal and luminal subtypes **A.** Hierarchical clustering was performed with the 15-miRNA signature in TCGA's cohort. (Left) Basal tumors identified by mRNA CC were isolated and clustered. The heatmap depicts relative expression of the 15-miRNA signature. (Right) Luminal tumors identified by mRNA CC were isolated and clustered. The heatmap depicts relative expression of the 15-miRNA signature. **B.** Hierarchical clustering with the 15-miRNA signature in the FF cohort. (Left) Basal tumors identified by mRNA PAM prediction were isolated and clustered. The heatmap depicts relative expression of the 15-miRNA signature. (Right) Luminal tumors identified by mRNA PAM prediction were isolated and clustered. The heatmap depicts relative expression of the 15-miRNA signature.

It was evident that a subpopulation of the basal tumors expressed lower levels of both basal and luminal miRNAs, and this subpopulation corresponded to p53-like tumors identified by the oneNN classifier. To explore this heterogeneity further, we utilized the class assignments from the TCGA 3-cluster (k = 3) mRNA CC solution to isolate the significant miRNAs and mRNAs that were differentially expressed by the p53-like “infiltrated” CC (Figure 4A). We identified 15 miRNAs by differential expression analysis and used this signature to perform hierarchical clustering with the basal or luminal tumors identified by mRNA CC. Overall, in the basal subtype, we isolated 82% of the infiltrated tumors identified by mRNA CC (k = 3), and in the luminal subtype we isolated 83% of the infiltrated tumors. The infiltrated luminal tumors identified by the 15-miRNA signature likely correspond to TCGA's cluster II, while the infiltrated basal tumors identified show similar expression patterns to TCGA's cluster IV tumors [5]. We applied the same 15-miRNA signature identified in TCGA's cohort to the 62 FF cohort. We isolated the basal and luminal tumors as identified by mRNA PAM, and performed hierarchical clustering with the 15-miRNA expression signature (Figure 4B). In the basal subtype, all of the infiltrated tumors identified by mRNA CC (k = 3) and oneNN p53-like tumors clustered together, while in the luminal subtype, 89% of the infiltrated tumors identified by CC were isolated by hierarchical clustering. The 15-miRNA signature includes miR-133b, mir-133a, mir-143, miR-145, miR-99a, and miR-100, which have been previously associated with fibrosis and chemo-resistance [33-35].

**Figure 5:**
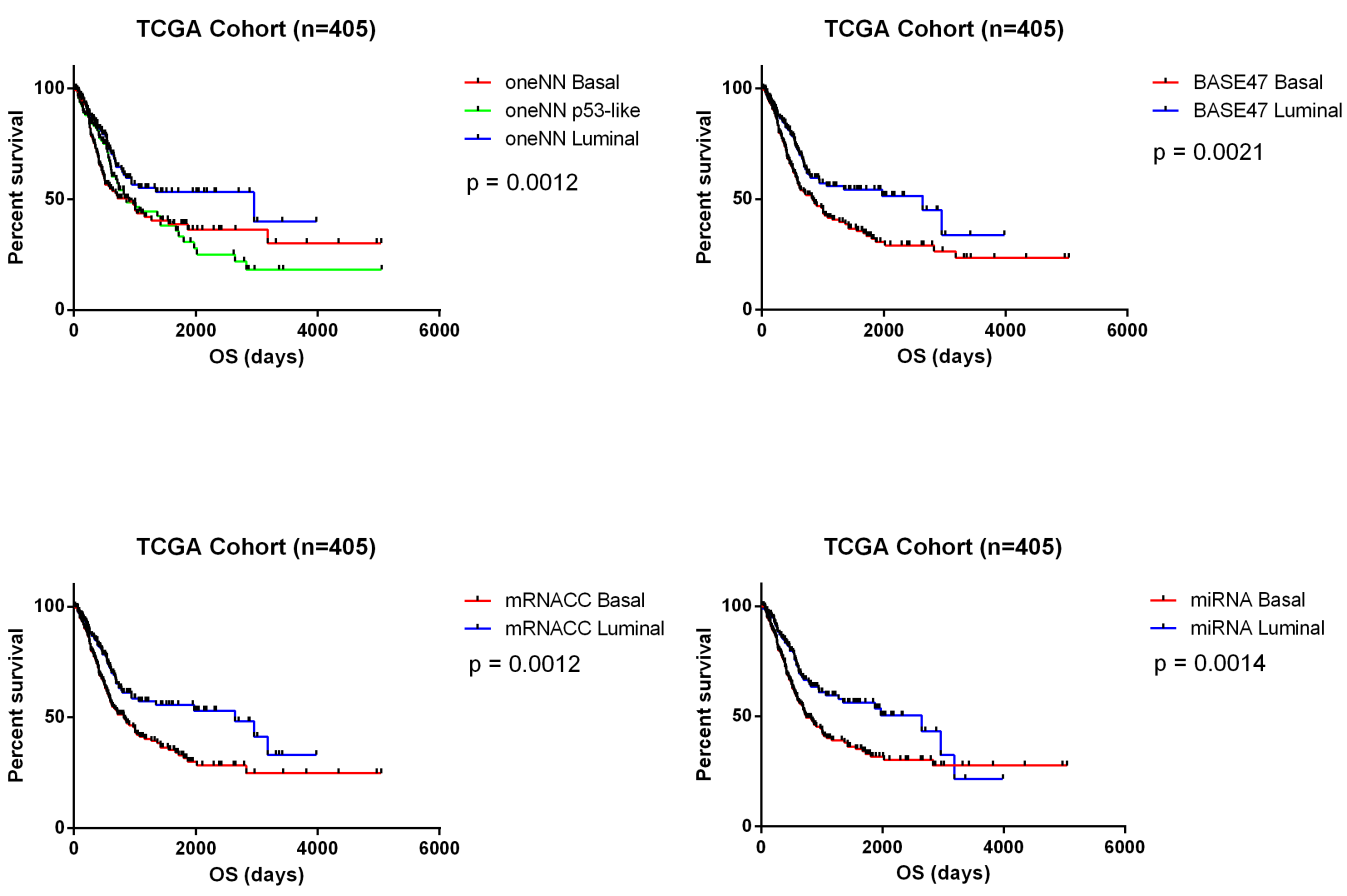
Survival analyses Survival analyses confirm that basal tumors have poor overall survival outcomes in TCGA's cohort (*n* = 405). **A.** Kaplan-Meier plot of overall survival based on MDA oneNN subtype assignments (*p* = 0.0012). **B.** Kaplan-Meier plot of overall survival based on BASE47 subtype assignments (*p* = 0.0021). **C.** Kaplan-Meier plot of overall survival based on mRNA CC subtype assignment (*p* = 0.0012). **D.** Kaplan-Meier plot of overall survival based on 63-miRNA signature assignments (*p* = 0.0014).

Lastly, we analyzed survival outcomes based on the subtype assignments made using the 63 PAM miRNAs in TCGA's cohort (*n* = 405). When we compared the 2-cluster solutions (BASE47, mRNA CC, 63-miRNA PAM signature), we observed that in all cases patients with basal tumors had the poorest clinical outcomes (Figure 5). We also analyzed TCGA's cohort after patients who received chemotherapy were removed, and saw similar survival outcomes (Figure S5). Having clinically available tests to prospectively identify these patients seems crucial, as our previous work showed that basal MIBCs responded well to platinum-based chemotherapy [14, 36].

## DISCUSSION

The primary goal of this study was to develop a miRNA-based classifier that could be used to assign tumors to the molecular subtypes of bladder cancer [4-6]. Using an unsupervised approach and a high quality RNAseq dataset, we reproduced our previous conclusion [14] that a 3-subtype solution was optimal mathematically. However, one of the subtypes, corresponding to our original p53-like subtype and consisting of MIBCs that were enriched with stromal biomarkers, was unstable [14, 36], and we therefore concluded that a two-subtype solution corresponded better with the known biology. Using CC and a k = 2 solution, we developed a new mRNA PAM classifier and compared the calls made using it to those made with BASE47. The results revealed over 90% concordance, consistent with the conclusion that the basal/luminal dichotomization is highly robust and most likely identifies the intrinsic subtypes of bladder cancer.

Although methods for producing high quality RNA expression data from some FFPE tissue sections are already available, the RNA in many of them is too degraded for whole transcriptome sequencing. Micro-RNAs are much more stable, so a miRNA-based subtype classifier would be expected to have a lower sample failure rate. In addition, miRNAs are also very stable in urine and blood, so it may be possible to perform tumor subtype calls using “liquid biopsies”. With these considerations in mind, we set out to develop a miRNA-based subtype classifier that could be used to accurately assign MIBCs to the basal and luminal subtypes. Using a supervised approach based on the mRNA subtype calls, we identified 63 miRNAs that were expressed in a largely mutually exclusive fashion in basal and luminal cancers. Analyses of their biological functions supported the idea that they should serve as robust biomarkers. Most of the basal miRNAs targeted factors that have been implicated in the control of luminal biology (FOXA1, ERBB2), adipogenesis (FGFR2, FGFR3) and urothelial differentiation (HOX5/8/13), all of which would be predicted to be suppressed in basal cancers. In contrast, the luminal miRNAs targeted transcription factors that controlled EMT (ZEB1/2), and biomarkers associated with fibrosis and actin cytoskeleton (collagens), and basal cancer biology (IL6, EGFR, STAT3), all of which are suppressed in luminal cancers. Using this 63-miRNA signature, we were able to accurately assign MIBCs to the basal and luminal subtypes and show that the basal tumors were associated with poor outcomes. Furthermore, we identified a 15-miRNA signature that identifies basal and luminal tumors that appear to be infiltrated with fibroblasts. This signature could prove useful in identifying tumors that are resistant to traditional chemotherapy [14, 34, 37, 38] but are sensitive to immune checkpoint blockade [3].

The high concordance of calls made using BASE47 and the new PAM classifier described here demonstrate that subtype calls made using relative mRNA expression are highly robust. Methods to quantify expression of large numbers of mRNAs isolated from FFPE tissues (Nanostring, RNAseq) are already available, so it should be possible to reduce these classifiers to clinical practice. However, RNA quality from FFPE tissues can be highly variable, and in our experience 40-50% of samples fail the standard quality control cut-off ( > 30% of RNA fragments longer than 200 bp) used for these platforms. In contrast, miRNAs are much more stable and can be reliably measured in highly degraded samples. Therefore, even though mRNA-based subtype calls appear to be somewhat more robust, a miRNA-based subtype classifier will have a lower sample failure rate. Importantly, we also recently created an immunohistochemical classifier based on KRT5/6 and GATA3 staining that can also distinguish basal and luminal tumors with reasonable accuracy [39]. Therefore, it seems likely that routine identification of basal and luminal tumors using mRNAs, miRNAs, or conventional immunohistochemistry will become part of routine diagnostic practice in the near future.

## MATERIALS AND METHODS

### Informed consent

Informed consent was obtained from all patients who contributed tumors to the TCGA and MD Anderson bladder cancer cohorts utilized in this study.

### Institutional review board (IRB) approval

All of the genomics studies (TCGA and MD Anderson) were performed in compliance with US guidelines under approved IRB laboratory protocols.

### Training dataset

BLCA RNA and miRNA-sequencing level 3 data was extracted from the TCGA data portal. TCGA normalized RNAseq (RSEM) and miRNA-Seq counts (RPM - reads per million mapped miRNA) were log2-transformed, median centered, and filtered based on a fold change of or greater than 2 in at least 10% of the samples. Messenger RNAs and miRNAs that passed the filtering criteria were used for consensus hierarchical clustering in the R package ConsensusClusterPlus [40], with 80% resampling and 1,000 iterations. Read counts for both mRNAs and miRNAs were used as input for differential expression analysis by the R package edgeR [41, 42]. All analyses utilized a false-discovery rate (FDR) cutoff of 5%, and a fold change cutoff of 2.

### Validation cohort

We utilized the MD Anderson 62 FF patient sample cohort that were analyzed by the Illuminia HTv3 beadchip microarray. The RNA expression data were downloaded from GEO, dataset GSE48075. The dataset was quantile normalized and differential expression was performed using the R Bioconductor package, linear models for microarray data (limma) [43].

### Subtype identification

Prediction analysis of microarrays (PAM) was used to identify the minimal number of mRNAs or miRNAs that could accurately predict subtype classification on TCGA's cohort using the mRNA CC (k = 2) calls as a reference [44]. The resulting 593-gene predictor (Δ = 6.969), and 63-miRNA predictor (Δ = 3.898), was used to classify the FF cohort.

### Ion torrent small RNA sequencing

We used 62 RNA samples to perform small RNA sequencing on the Ion Proton from Ion Torrent. The amount of small RNA in the total RNA sample was quantified with the use of Small RNA and RNA 6000 Nano bioanalyzer chips from Life Technologies. We used 20 ng of small RNA for library preparation using the Ion Total RNA Seq v2 library preparation kit. The resulting cDNA library was quantified with High Sensitivity DNA bioanalyzer chips from Life Technologies to determine the molar concentration of each library, and to calculate the percentage of library that was barcoded small RNAs. The cDNA libraries were diluted to the same molar concentration, pooled, and diluted to 100 pM. The sample was then templated and sequenced with the Ion Proton. The GEO accession number for the small RNA sequencing data presented in this study is GSE84525.

### Identification of downstream targets

In order to identify downstream target mRNAs of the identified differentially expressed miRNAs, we used the IPA (Ingenuity Systems) miRNA target filter to identify potential downstream mRNA targets. Target mRNAs that were high in a subset, where the miRNA was low in the same subset were isolated and used for analyses.

### Survival analyses

Clinical data for the 405 TCGA patient samples was extracted from TCGA's data portal. All survival analyses were performed in GraphPad Prism 6.

## SUPPLEMENTARY MATERIALS


