# Supplementary material for: Specific micro-RNA expression patterns distinguish the basal and luminal subtypes of muscle-invasive bladder cancer
Source: Oncotarget. 2016 Nov 10;7(49):80164–74. doi: 10.18632/oncotarget.13284 (PMC5348311; doi:10.18632/oncotarget.13284)
Supplement: Supplementary file 1 [file oncotarget-07-80164-s001.pdf]

Specific micro-RNA expression patterns distinguish the basal and luminal subtypes of muscle-invasive bladder cancer

Supplementary Material

A.

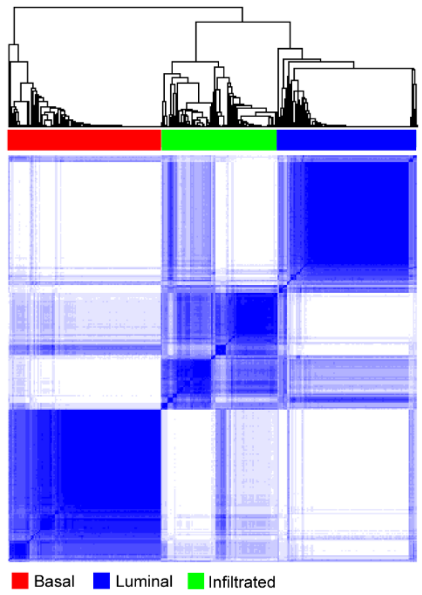

B.

**CC Basal**		
	oneNN Basal	**124**
	oneNN Luminal	**11**
	oneNN p53-like	**16**
**CC Luminal**		
	oneNN Basal	**4**
	oneNN Luminal	**109**
	oneNN p53-like	**25**
**CC Infiltrated**		
	oneNN Basal	**9**
	oneNN Luminal	**35**
	oneNN p53-like	**72**

**CC Basal**		
	BASE47 Basal	**145**
	BASE47 Luminal	**6**
**CC Luminal**		
	BASE47 Basal	**6**
	BASE47 Luminal	**132**
**CC Infiltrated**		
	BASE47 Basal	**49**
	BASE47 Luminal	**67**

C.

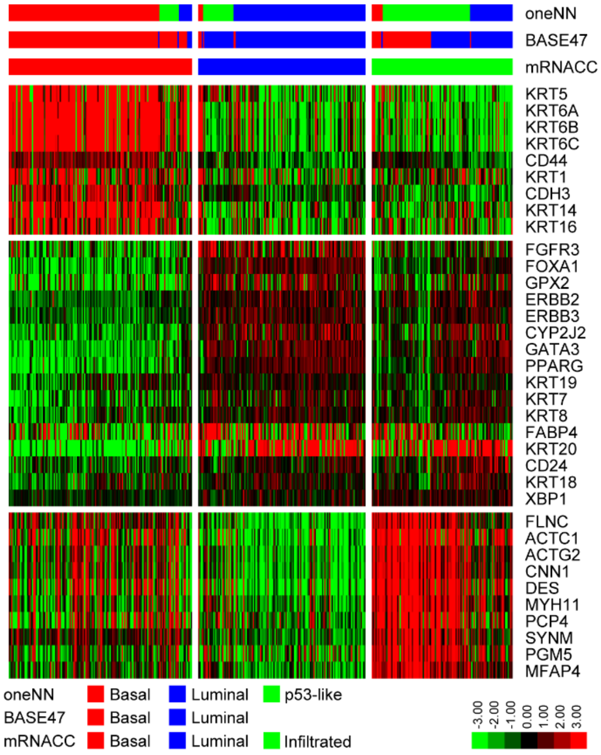

D.

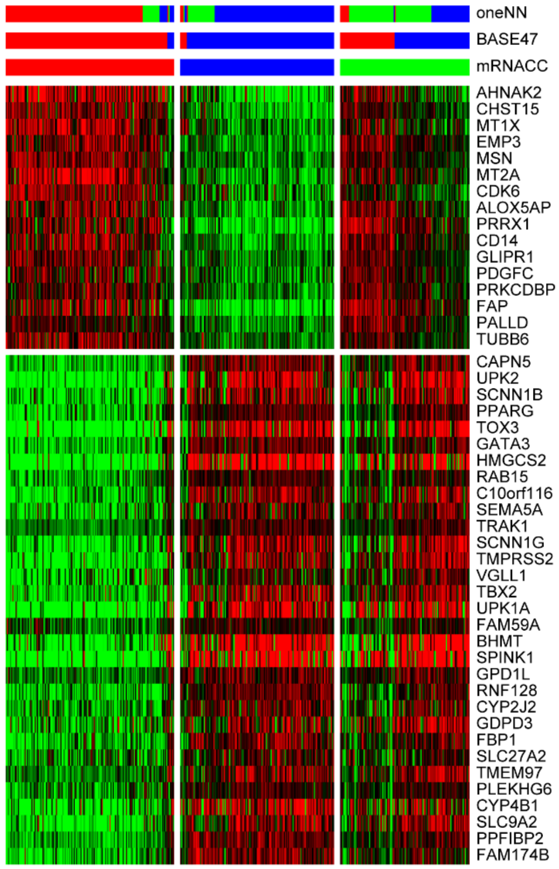

Supplementary Figure S1: mRNA CC 3-cluster solution (k=3) with TCGA's cohort (n=405). A. mRNA CC of TCGA's 405 MIBCs identified 3 distinct clusters. B.

Comparison of mRNA CC subtype assignments to MDA oneNN assignments (left), and to BASE47 assignments (right). C. The heatmap depicts relative expression of MDA basal (top) and luminal (bottom) markers. D. The heatmap depicts relative expression of BASE47 basal (top) and luminal (bottom) markers.

Characteristics of the MDA validation Cohort (n=62)

Supplementary Table S1: Clinicopathologic characteristics of the MDA validation cohort (n=62). The Mann-Whitney U test was used to compare differences in mean age between groups. The log-rank test was used to compare differences in survival between groups. Fisher's exact test was used to determine differences between groups for the remainder of categorical variables.

	Total	Basal	Luminal	p-value
Cohort Size (n)	62	28 (45%)	34 (55%)	
Mean Age (y) ± SD	68.7 ± 9.9	69.8 ± 10.3	67.9 ± 9.6	0.686
Clinical Stage at Specimen Collection (n)				
cT2	38 (61%)	17 (61%)	21 (62%)	0.774
cT3	18 (29%)	9 (32%)	9 (26%)	
cT4	6 (10%)	2 (7%)	4 (12%)	
Positive Clinical Lymph Nodes (n)	10 (16%)	5 (18%)	5 (15%)	0.744
Positive Clinical Metastasis (n)	5 (8%)	3 (11%)	2 (6%)	0.65
Median Overall Survival (m)	46.30	18.70	82.40	0.073

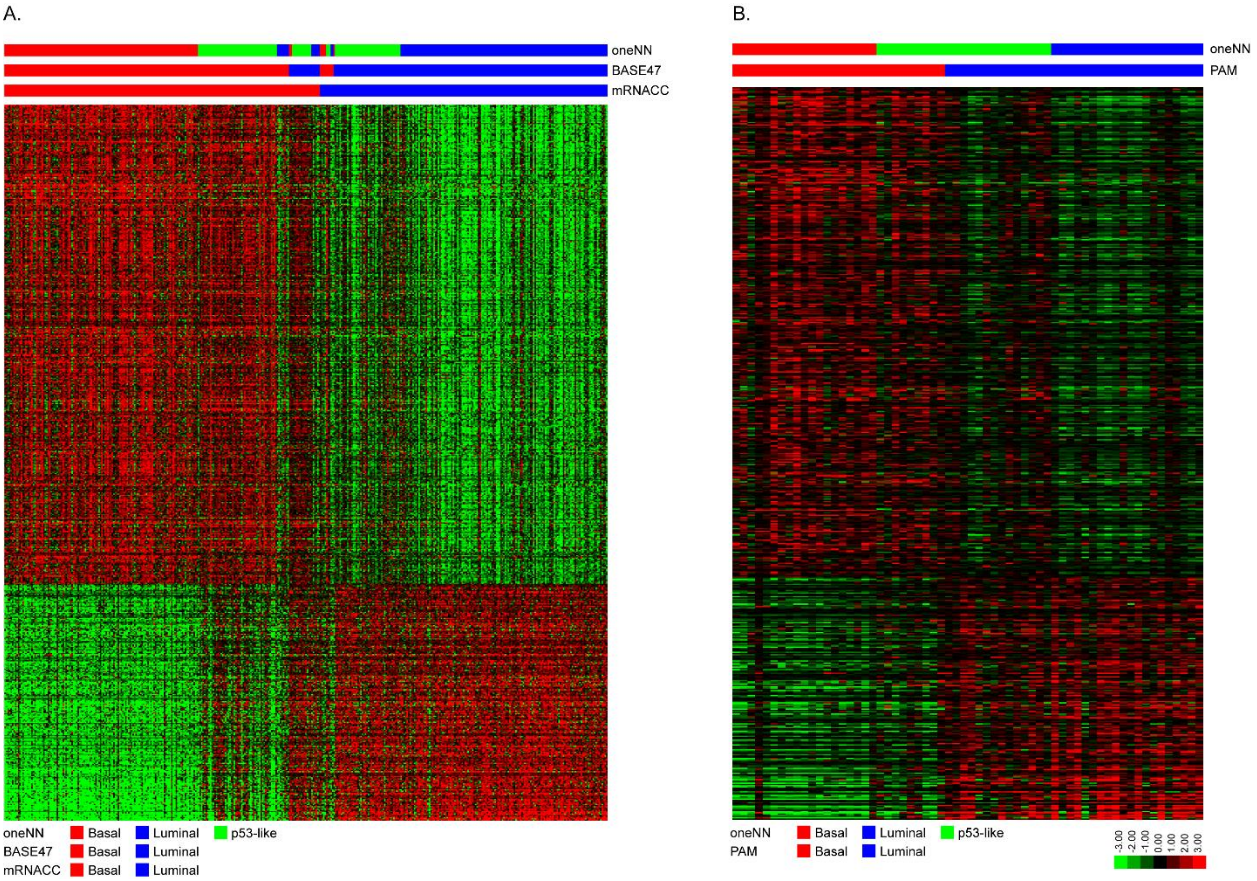

Supplementary Figure S2: 593-mRNA PAM genes. A. The heatmap depicts relative expression of the 593-mRNA PAM genes in TCGA's cohort. B. The heatmap depicts relative expression of the 593-mRNA PAM genes in the FF cohort.

A.

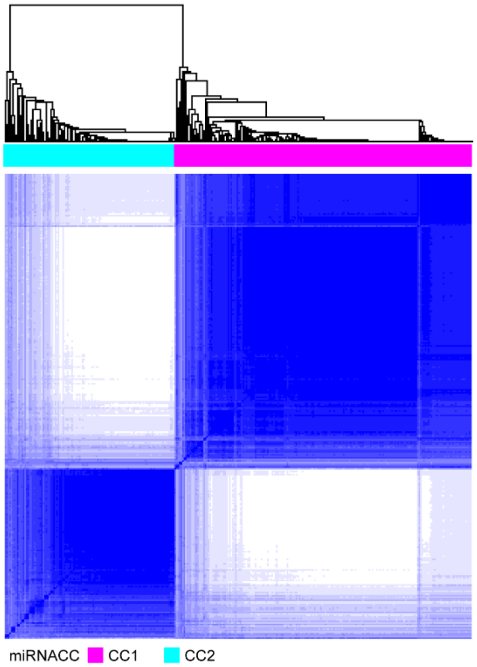

B.

miRNACC1		
	CC basal	188
	CC luminal	69
miRNACC2		
	CC basal	24
	CC luminal	124
miRNACC1		
	BASE47 basal	174
	BASE47 luminal	83
miRNACC2		
	BASE47 basal	26
	BASE47 luminal	122
miRNACC1		
	oneNN basal	124
	oneNN p53-like	86
	oneNN luminal	47
miRNACC2		
	oneNN basal	13
	oneNN p53-like	27
	oneNN luminal	108

C.

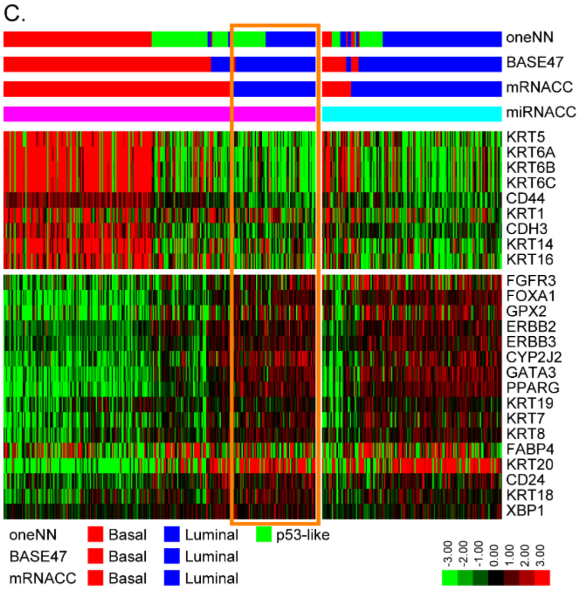

D.

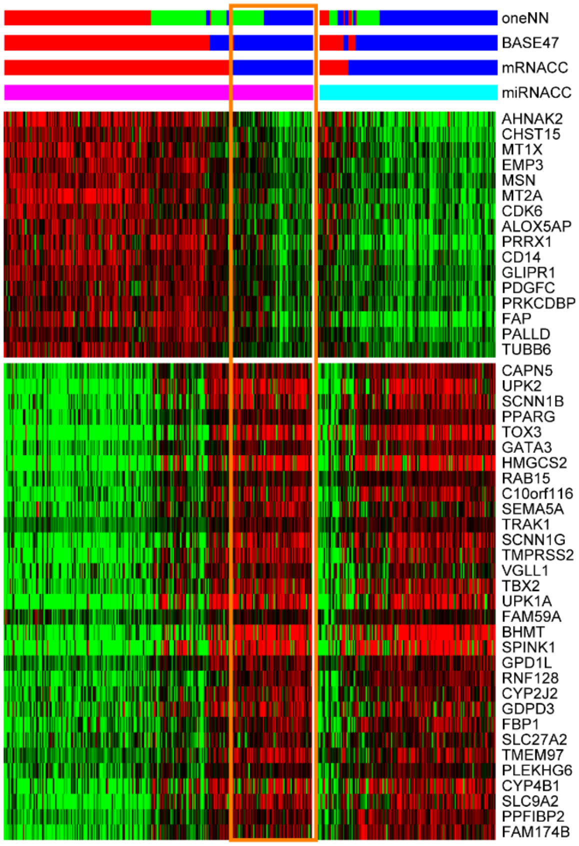

Supplementary Figure S3: Micro-RNA CC (k=2) was performed with TCGA's cohort (n=405). A. Micro-RNA CC identified 2 clusters in TCGA's cohort. B. Comparison of miRNA CC subtype assignments to mRNA CC assignments (top), to BASE47 assignments (middle), and to MDA oneNN assignments (bottom). C. The heatmap depicts relative expression of MDA basal (top) and luminal (bottom) markers. D. The heatmap depicts relative expression of BASE47 basal (top) and luminal (bottom) markers. Note: orange boxes indicate luminal tumors that were misclassified.

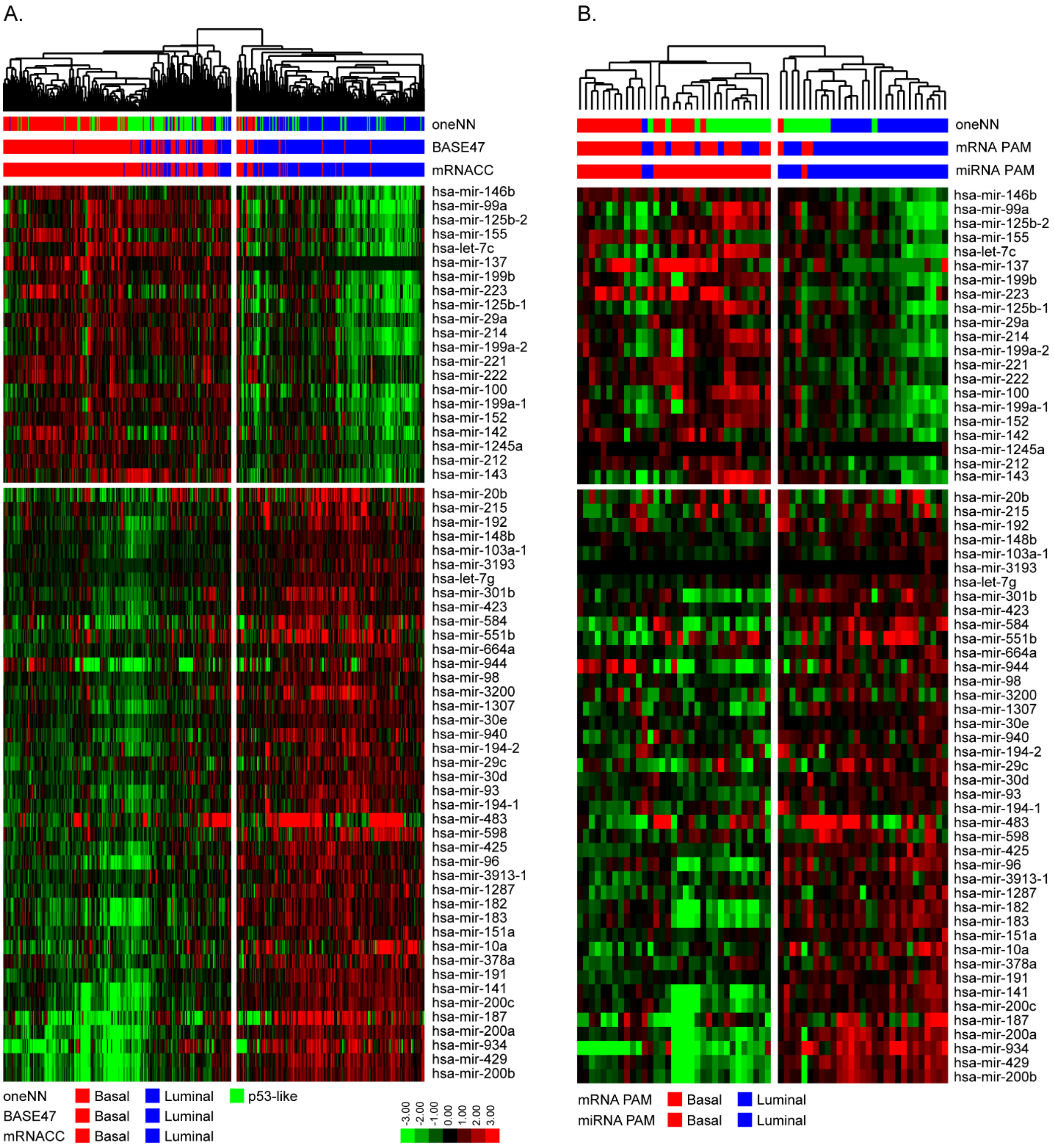

Supplementary Figure S4: Hierarchical clustering with the 63 miRNAs identified by PAM A. Hierarchical clustering was performed with the 63-miRNA signature in TCGA's cohort. The heatmap depicts relative expression of the 63 miRNAs. B. Hierarchical clustering with the 63-miRNA signature in the FF cohort. The heatmap depicts relative expression of the 63 miRNAs.

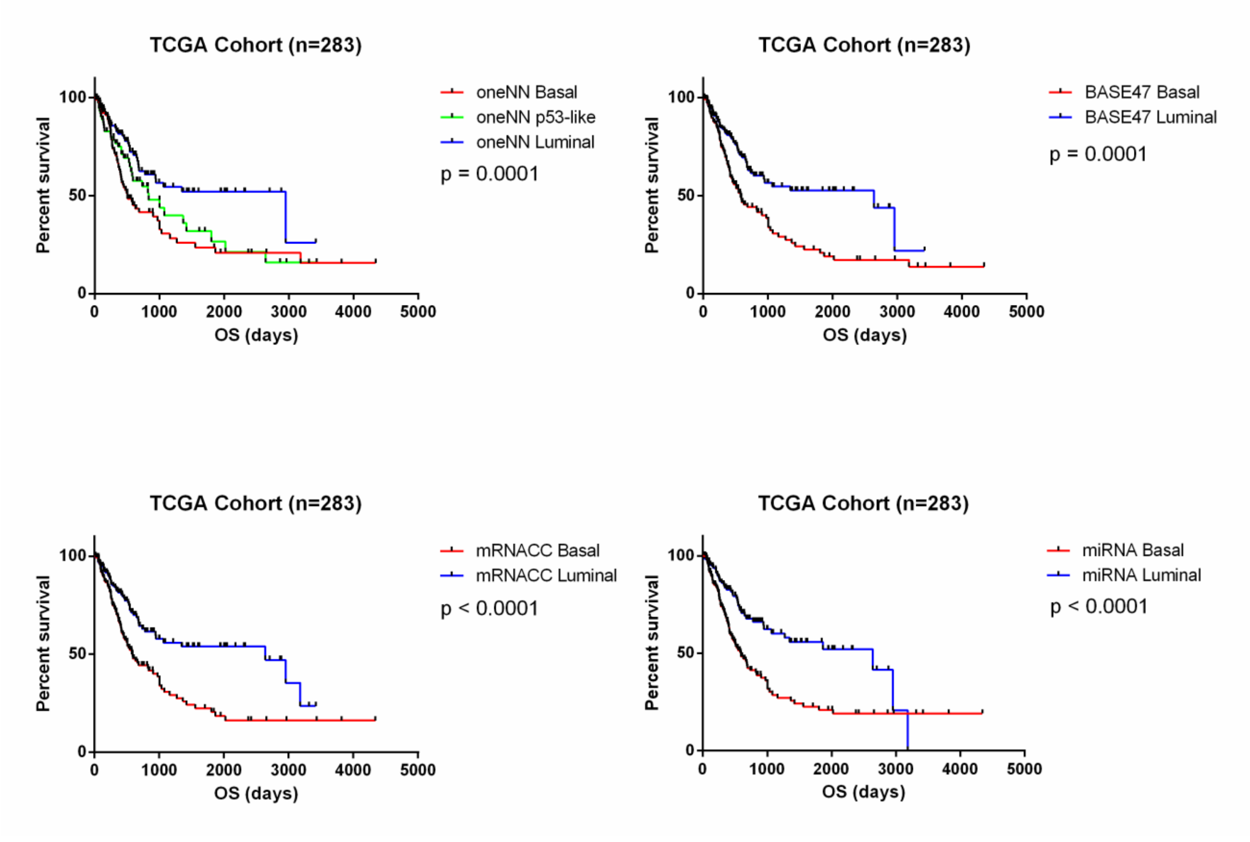

Supplementary Figure S5: Survival analyses. Survival analyses confirm that patients with basal tumors, in the absence of chemotherapy, have poor overall survival outcomes. A. Kaplan-Meier plot of overall survival based on MDA oneNN subtype assignments (p=0.0001). B. Kaplan-Meier plot of overall survival based on BASE47 subtype assignments (p=0.0001). C. Kaplan-Meier plot of overall survival based on mRNA CC subtype assignment ($p < 0.0001$). D. Kaplan-Meier plot of overall survival based on the 63 PAM miRNA signature assignments ($p < 0.0001$).
